# Predicting the Potential Distribution of *Aconitum carmichaelii* Debeaux in China Under Climate Change Using an Optimized MaxEnt Model

**DOI:** 10.3390/plants15071067

**Published:** 2026-03-31

**Authors:** Jieru Chen, Wei Zhang, Shimeng Cui, Xinyue Zhu, Yangyang Chen, Jingyuan Ren, Ziling Liu, Yiqiong Liu, Hai Liao, Jiayu Zhou

**Affiliations:** 1 School of Life Science and Engineering, Southwest Jiaotong University, Chengdu 610031, China; chenjieru0617@163.com (J.C.); 18280485652@163.com (W.Z.); 13684406357@163.com (S.C.); xzzb3458@163.com (X.Z.); cyysicnu@163.com (Y.C.); jingyuanren2003@163.com (J.R.); 18398010183@163.com (Z.L.); 2 Chengdu Botanical Garden, Chengdu 610083, China

**Keywords:** *Aconitum carmichaelii* Debeaux, climatic scenarios, distribution migration, model optimization, sustainable utilization

## Abstract

*Aconitum carmichaelii* Debeaux has been a traditional medicinal resource in China for over two millennia. However, sustainable utilization and preservation strategies for *A. carmichaelii* require a thorough understanding of environmental factors influencing its distribution. An optimized MaxEnt model was constructed using the ENMeval package based on 185 quality-controlled occurrence records and 10 selected environmental variables (bioclimatic, edaphic, topographic, and anthropogenic). The optimized model demonstrated reliable predictive accuracy, with an area under curve (AUC) value of 0.896. Soil moisture (37.7% contribution), human footprint (HFP) (23.9%), and July solar radiation (11.1%) were the primary variables determining *A. carmichaelii* distribution. The suitable thresholds were defined as soil moisture > 87.34 mm, HFP > 10.69, and July solar radiation < 19,125.72 kJ m^−2^ day^−1^. At present, highly suitable habitat covers approximately 8.243 × 10^5^ km^2^, predominantly located in the Sichuan Basin and surrounding regions, including Sichuan, Chongqing, Guizhou, and northeastern Yunnan. Future predictions under all Shared Socioeconomic Pathway (SSP) scenarios indicate a significant reduction in highly suitable habitat, with losses of 63.01% (2041–2060, SSP126), 62.62% (2041–2060, SSP245), 61.35% (2041–2060, SSP370), and 61.99% (2061–2080, SSP585). Habitat contraction mainly occurs toward higher altitudes and southwestern areas, with a maximum displacement distance of 50.56 km under the SSP585 scenario. This study enhances our understanding of environmental factors affecting the distribution of *A. carmichaelii* and offers guidance for its sustainable management and cultivation amid global climate change.

## 1. Introduction

Under four Shared Socioeconomic Pathway (SSP) scenarios, global mean temperatures are projected to increase by 1.8–4.4 °C by 2100 compared with 1850–1880 [1]. In Southwest China, the complex topography of the Sichuan Basin and the Yunnan-Guizhou Plateau creates a mosaic of microclimates. These microclimates effectively buffer the region against severe macroclimatic fluctuations [2]. The climatic characteristics of this area are predominantly influenced by the East Asian and Indian summer monsoons, which maintain stable soil moisture levels [3], crucial for plant physiological persistence. Additionally, significant altitudinal gradients facilitate the formation of specialized ecological niches, making Southwest China one of the world’s most critical biodiversity hotspots [4]. Nevertheless, the combined effects of climate-driven habitat contraction and intensive human activities in these densely populated regions pose substantial challenges for the sustainable management of wild plant resources [5]. Given that climate change profoundly reshapes species’ spatial distributions [6], quantifying the extent of such influences is essential for developing conservation strategies [7].

The genus *Aconitum* L. (Ranunculaceae) comprises over 160 species in China, many historically recognized as valuable medicinal herbs [8,9]. Among them, *Aconitum carmichaelii* Debeaux is the most extensively cultivated and utilized species in Traditional Chinese Medicine (TCM), with a history spanning over 2000 years and is regarded as a representative medicinal plant in southwest China [10]. To date, the continuously expanding market demand for *A. carmichaelii* has driven large-scale artificial cultivation. Although cultivation practices alleviate pressure on wild resources and ensure a sustainable supply, systematic and science-based guidance for current agricultural methods remains inadequate. As China represents a major biodiversity hotspot for *Aconitum* species, it provides an ideal region to examine the habitat suitability of *A. carmichaelii*. However, the environmental variables shaping the distribution and future potential distribution of *A. carmichaelii* remain unexplored. Addressing these knowledge gaps has become crucial for its conservation and sustainable utilization.

To resolve these spatial challenges, species distribution models (SDMs) have been widely employed to investigate the response of suitable habitat to environmental changes [7]. Among these methods, the maximum entropy (MaxEnt) model is particularly effective due to its reliable predictive performance, especially with presence-only data and complex nonlinear relationships [11,12]. The MaxEnt model also shows exceptional algorithmic stability across varying sample sizes, from limited to extensive occurrence records. Despite its popularity, routine applications of MaxEnt with default software settings often result in severe model overfitting and poor transferability to future climatic scenarios [13]. Thus, parameter optimization (e.g., tuning regularization multipliers and feature classes) is essential for generating biologically meaningful and mathematically accurate predictions.

The MaxEnt model has recently been applied to studies on plants in the family Ranunculaceae [14,15,16], but no comprehensive analysis of the suitable habitat for *A. carmichaelii* using an optimized MaxEnt model has been conducted. The primary objectives of this study were to: (1) construct an ENMeval-optimized MaxEnt model; (2) determine the dominant variables influencing the distribution of *A. carmichaelii*; (3) simulate suitable habitats under current and future climatic scenarios using the optimized MaxEnt model; and (4) predict future distributional trends under divergent climatic scenarios. These findings aim to provide a rigorous scientific foundation for optimizing cultivation layouts, refining agrotechnical systems, and formulating conservation strategies for *A. carmichaelii* in response to global climate change.

## 2. Results

### 2.1. Optimal Model and Model Evaluation

**Figure 1:**
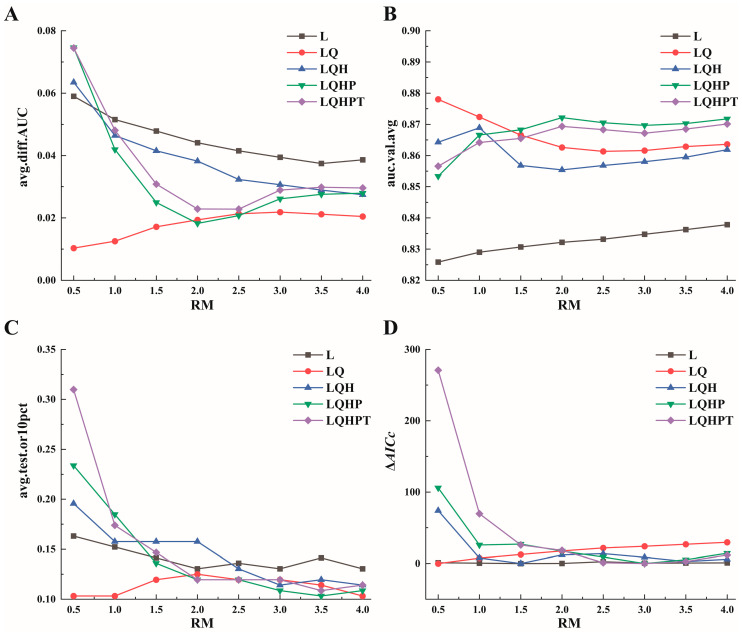
Variation in avg.diff.AUC (**A**), auc.val.avg (**B**), avg.test.or10pct (**C**), and Δ*AICc* (**D**) parameters during model optimization.

Under default settings (regularization multiplier (RM) = 1; feature combinations (FC) = LQHPT), the initial MaxEnt model yielded a minimum corrected Akaike Information Criterion (Δ*AICc*) of 69.71, an average AUC difference (avg.diff.AUC) of 0.0481, a 10% training omission rate (avg.test.or10pct) of 0.1739, and an average/sd AUC calculated on the validation datasets (auc.val.avg) of 0.804. After optimization, the final MaxEnt model adopted parameters RM = 0.5 and FC = LQ, achieving optimal model parsimony with a Δ*AICc* of 0. Compared to the default-parameter MaxEnt model, the optimized model showed significantly improved predictive performance. Specifically, the avg.diff.AUC, avg.test.or10pct decreased by 21.45%, 59.4% while the auc.val.avg increased by 3.48%, respectively (Figure 1). These optimized parameters effectively controlled model complexity and enhanced transferability for subsequent habitat suitability predictions.

**Figure 2:**
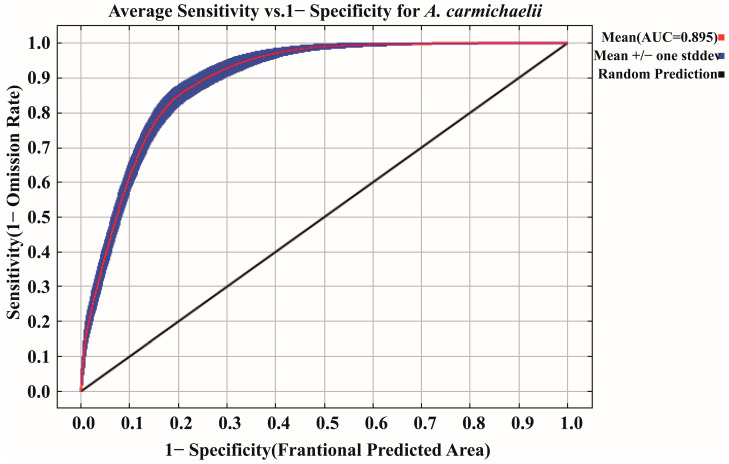
AUC assessment of regularized training for *A. carmichaelii*.

Model performance varied considerably across different data-split ratios, reflected by changes in Area Under the Curve (AUC) values (Figure 2). By systematically evaluating these metrics, the optimal data-split strategy was identified to maximize model robustness (Table 1). Consequently, 75% of occurrence records were used as the training set for model calibration, and 25% were reserved as the test set for independent evaluation and validation.

### 2.2. Importance of Environmental Variables in Determining the Distribution of A. carmichaelii

**Figure 3:**
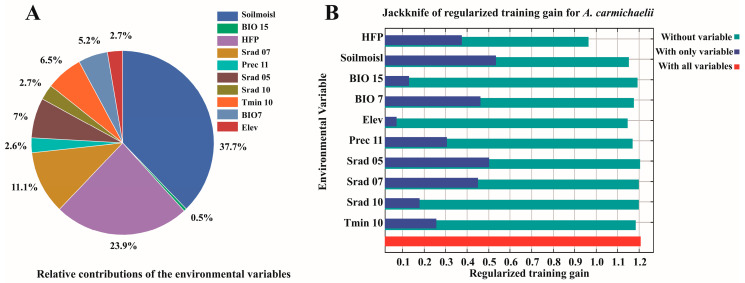
Evaluation of environmental variables in the MaxEnt model. (**A**) Relative contribution of each environmental variable; (**B**) Jackknife test of environmental variables to regularized training gain.

**Figure 4:**
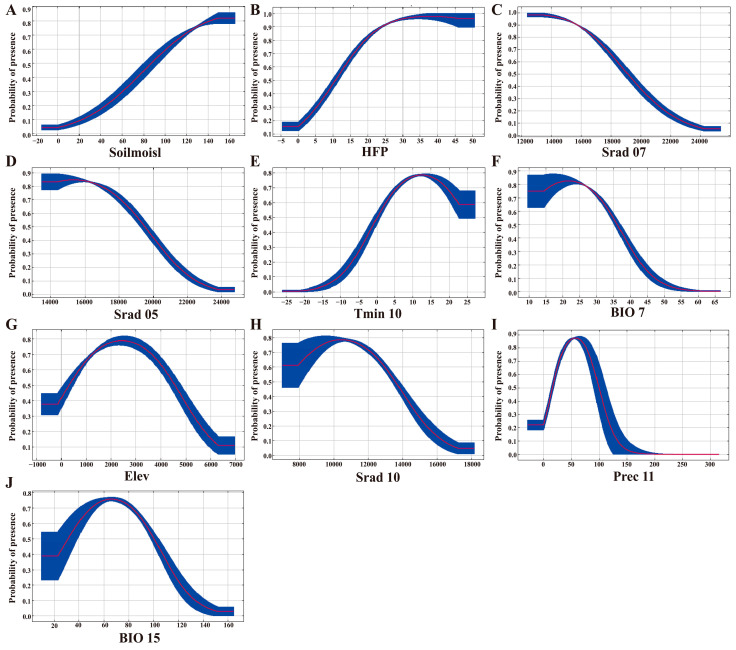
Response curves of environmental variables in the MaxEnt model for *A. carmichaelii*. (**A**–**J**) represent the response curves for soil moisture, HFP, Srad 07, Srad 05, Tmin 10, BIO 7, Elev, Srad 10, Prec 11, and BIO 15, respectively. The curves show the mean response of 10 replicate MaxEnt runs (red) ± one standard deviation (blue).

Analysis of variable importance within the optimized MaxEnt model identified soil moisture (37.7%) as the most influential environmental variable. It was followed by the human footprint (HFP, 23.9%), solar radiation in July (Srad 07, 11.1%), solar radiation in May (Srad 05, 7.0%), minimum temperature in October (Tmin 10, 6.5%), and temperature annual range (BIO 7, 5.2%). Together, these six variables accounted for 91.4% of the cumulative contribution (Figure 3A), highlighting their critical roles in shaping the geographic distribution of *A. carmichaelii*. Results from the jackknife test further supported the contribution of these variables (Figure 3B). Response curve represents the correlation between the environmental variable and the probability of the presence of *A. carmichaelii*. As a result, response curves (Figure 4) indicated suitable conditions for *A. carmichaelii* included soil moisture above 87.34 mm, HFP values greater than 10.69, Srad 07 and Srad 05 below 19,125.72 kJ m^−2^ day^−1^ and 19,513.91 kJ m^−2^ day^−1^, respectively, and Tmin 10 above 0.1184 °C. BIO 7 showed suitability below 36.05 °C, with the highest presence probability at 21.71 °C. Variables such as altitude (Elev), solar radiation in October (Srad 10), November precipitation (Prec 11), and precipitation seasonality (coefficient of variation) (BIO 15) were less significant, with suitable ranges of 313–4563 m, below 13,577.05 kJ m^−2^ day^−1^, 16.42–99.77 mm, and 31.24–100.36, respectively.

### 2.3. Predicted Distribution of A. carmichaelii Under Current and Future Climatic Scenarios

**Figure 5:**
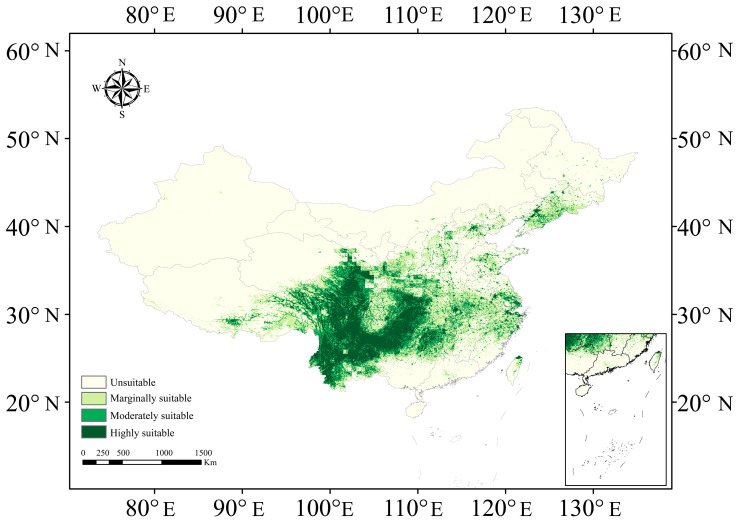
Predicted current distribution of *A. carmichaelii* in China. Suitability levels: highly suitable (0.6–1.0), moderately suitable (0.4–0.6), marginally suitable (0.2–0.4), and unsuitable (0–0.2).

The optimized MaxEnt model aligned relatively high spatial congruence with current *A. carmichaelii* distribution records (Figure 5). Currently, highly suitable habitat covers 8.243 × 10^5^ km^2^ (8.6% of the study area), primarily concentrated in the Sichuan Basin and Yunnan-Guizhou Plateau. These regions are influenced by the East Asian monsoon, which maintains stable and sufficient soil moisture levels required by *A. carmichaelii*. Moderately suitable and marginally suitable habitats occupy 7.048 × 10^5^ km^2^ (7.35%) and 12.422 × 10^5^ km^2^ (12.96%), respectively. Unsuitable area encompasses 68.127 × 10^5^ km^2^ (71.08%) (Table 2). Geographically, highly suitable habitat clusters in the Sichuan Basin and its periphery, encompassing most of Sichuan, western Chongqing, northern Guizhou, and parts of northeastern Yunnan and southern Shanxi. The highly suitable habitat is bordered by moderately suitable habitat extending into Hunan, Jiangxi, and southern Hubei. Marginally suitable habitat is distributed along the periphery (Figure 5).

**Figure 6:**
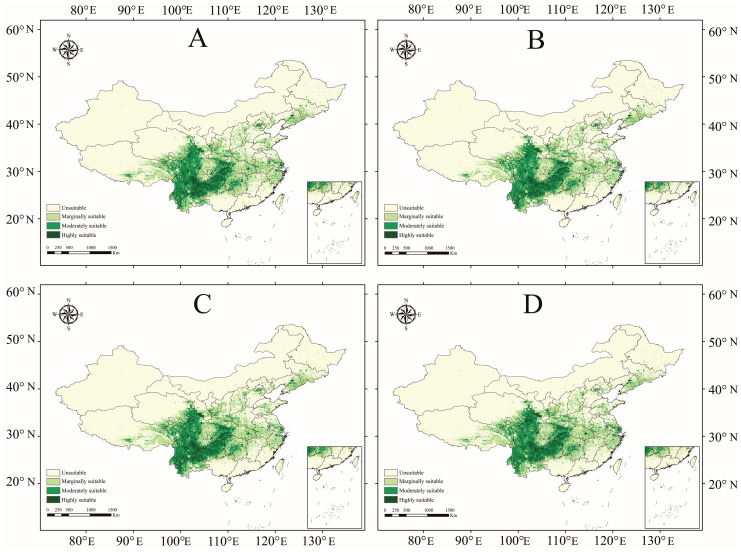
The predicted distribution of *A. carmichaelii* for the period 2021–2040 under four climatic scenarios: (**A**) SSP126, (**B**) SSP245, (**C**) SSP370, and (**D**) SSP585.

Future predictions (Table 2, Figure 6 and Figure S1) indicated significant habitat contraction for *A. carmichaelii* under all climatic scenarios relative to the current baseline (8.243 × 10^5^ km^2^). Under SSP126, the highly suitable habitat shows multiphasic fluctuation: declining initially to 3.220 × 10^5^ km^2^ (2021–2040), slightly reducing to 3.049 × 10^5^ km^2^ (2041–2060), rebounding to 3.810 × 10^5^ km^2^ (2061–2080), and declining again to 3.333 × 10^5^ km^2^ by 2100. Under SSP245, highly suitable habitat remains relatively stable, fluctuating between 3.0 and 3.2 × 10^5^ km^2^. Scenarios with higher greenhouse gas emissions (SSP370 and SSP585) exhibit late-century peaks. Under SSP370, suitable habitat initially expands slightly, reaching 3.548 × 10^5^ km^2^ (2061–2080) before declining. The SSP585 scenario follows a decline-increase trend, recovering from early losses to 3.438 × 10^5^ km^2^ (2081–2100). Overall, highly suitable habitat areas experience substantial losses, retaining less than half their current extent.

### 2.4. Centroid Shifts of Suitable Habitats

**Figure 7:**
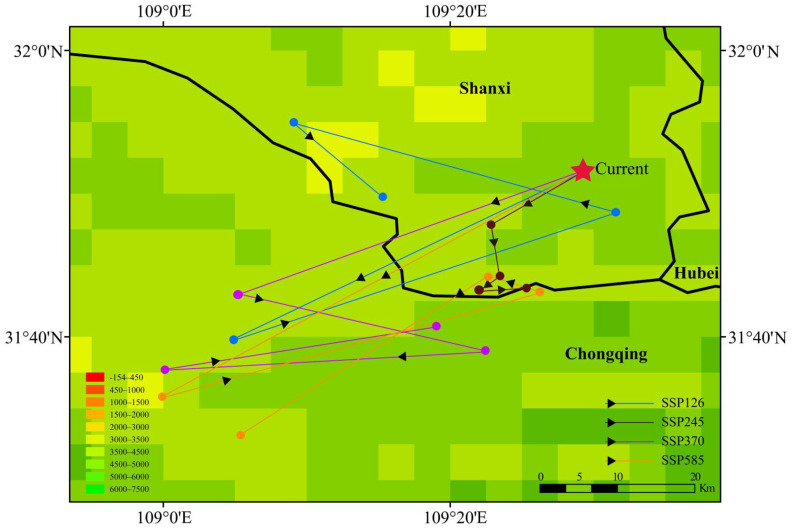
Centroid migration of *A. carmichaelii* under future climatic scenarios (SSP126, SSP245, SSP370, and SSP585).

Spatial centroid analysis of suitable habitats was performed using ArcGIS (version 10.8) to track geographical shifts under future climate scenarios (Figure 7). Currently, the centroid is located at the administrative junction of Shanxi, Hubei, and Chongqing. Under SSP126, the centroid migrates westward to southwestern Shanxi (109.255° E, 31.829° N), shifting by 23.74 km. Under SSP245, SSP370, and SSP585 scenarios, centroids shift southwestward to the junction of Shaanxi and Chongqing (109.422° E, 31.723° N), northern Chongqing (109.318° E, 31.679° N), and northern Chongqing (109.09° E, 31.552° N), respectively, with migration distances of 15.34 km, 25.09 km, and 50.56 km. These findings illustrate systematic spatial reconfiguration of *A. carmichaelii* habitats, with migration intensity increasing alongside radiative forcing. Higher-emission scenarios cause pronounced geographical displacement toward higher elevations or buffered microclimates in southwestern regions.

## 3. Discussion

### 3.1. Optimization Performance of MaxEnt

To accurately reflect the current habitat conditions, occurrence records collected after the year 2000 were selected. Using 185 occurrence records and 10 environmental variables, the MaxEnt model was optimized to predict the potential distribution of *A. carmichaelii* under current and future climatic scenarios. Default MaxEnt parameters frequently lead to model overfitting and excessive complexity, reducing interpretability and prediction accuracy. Therefore, the ENMeval package was employed to optimize model parameters. By adjusting the regularization multiplier (RM) from 1.0 to 0.5 and simplifying FC from LQHPT to LQ, the Δ*AICc* was reduced from 69.71 to 0, significantly minimizing overfitting and producing a more parsimonious model. The resulting AUC of 0.895 indicates reliable predictive performance and favorable discriminative ability. Additionally, the predicted highly suitable habitat, primarily concentrated in Sichuan, Yunnan, and Chongqing, closely aligns with the known range of *A. carmichaelii*, further validating the reliability of the constructed model.

### 3.2. Importance of Environmental Variables

The predicted distribution of *A. carmichaelii* is influenced by multiple environmental variables, with soil moisture being the most critical (37.7% contribution). The suitable threshold identified for soil moisture is 87.34 mm. Regarding its ecological characteristics, humid environment is suitable for *A. carmichaelii* [17]. Adequate soil moisture is essential for sustaining physiological metabolism [18,19]; for example, photosynthetic efficiency in *A. carmichaelii* is sensitive to nitrogen (N) and phosphorus (P) enrichment and drought stress mitigation [20]. Moreover, optimal water supply improves rhizosphere soil properties and microbial community composition, further enhancing plant yield [21,22,23]. Concurrently, the HFP is the second most significant variable (23.9% contribution), emphasizing the impact of human activities on the species’ distribution. Previous studies have demonstrated that human activities significantly affect environmental quality and biodiversity [5]. Additionally, human activity contributes to the spatial distribution of other species, such as *Danaus genutia* (Cramer) and *Aconitum leucostomum* Vorosch. [24,25]. However, Xu et al. [24] indicated that HFP inhibited the spread of *A. leucostomum*, which is inconsistent with our result. Considering that *A. leucostomum* is a kind of poisonous grass, it is inferred that HFP is conducive to reduce the damage of *A. leucostomum* on the grassland and financial income of local herders, while *A. carmichaelii* is likely to benefit from HFP due to its high economic values. Furthermore, potential sampling bias or the inadvertent inclusion of cultivated *A. carmichaelii* populations may partly account for the high contribution of the HFP variable. This underscores the necessity for more rigorous screening of wild occurrence records.

Solar radiation is considered to play role in many aspects in plant growth and developments, including biomass accumulation [26], flowering [27] and photosynthesis [28], however, excessive energy of solar irrandiance causes damage to many cellular components [28]. May (Srad 05) and July (Srad 07) represent periods of vigorous vegetative growth. Both variables show a clear negative correlation with suitability level, indicating that excessive solar radiation during these stages impairs photosystem II (PSII) activity and induces oxidative stress [29]. Similarly, October represents a critical transition period when energy is translocated to lateral roots for bioactive alkaloid accumulation. A stable minimum temperature (Tmin 10) above 0.1184 °C and moderate solar radiation (Srad 10) might prevent premature frost damage and maximize root biomass before winter dormancy. Additionally, a narrow annual temperature range (BIO 7 < 36.05 °C) is implied to be essential for perennial survival and long-term population persistence, suggesting that *A. carmichaelii* favors climatic stability over variability [30].

Finally, BIO 15, Srad 10, Prec 11, and Elev serve as secondary constraints. November (Prec 11) marks the onset of deep dormancy; excessive precipitation during this period potentially increases the risk of fatal root rot [31]. Intriguingly, although typically regarded as a species found at low and medium elevations, *A. carmichaelii* exhibits a broad altitudinal range (313–4563 m). Thus, *A. carmichaelii* may be cultivated across diverse elevations, potentially expanding cultivation zones under future climate scenarios.

### 3.3. Dynamic Shifts in Suitable Habitats of A. carmichaelii

Global climate change has profoundly altered species’ spatial distribution patterns, particularly affecting ecologically sensitive medicinal taxa [32]. Current predictions indicate that highly suitable habitat for *A. carmichaelii* under all four SSP scenarios will decrease by more than 50% compared to the current area (8.243 × 10^5^ km^2^). This significant reduction underscores the acute sensitivity of *A. carmichaelii* to environmental disturbances, consistent with Xiao et al. [17], who noted the species’ preference for relatively low temperatures (mean annual temperature < 15 °C). Zhang et al. [12] also reported a similar decline in suitable habitats with rising greenhouse gas emissions, aligning with the established ecological paradigm that climate-driven changes exacerbate habitat fragmentation and range contraction in endemic species [30,33]. As the dominant determinant (soil moisture, 37.7% contribution) of *A. carmichaelii* distribution, stable soil moisture supports the species’ presence in humid regions like the Sichuan Basin and Yunnan-Guizhou Plateau. However, predicted temperature increases and subsequent elevated evapotranspiration may surpass the critical threshold (87.34 mm) for the species, ultimately causing a decline in highly suitable habitat due to intensified drought stress [33].

The habitat centroid of *A. carmichaelii* demonstrates a general migration trajectory toward the west and southwest, with the largest displacement (50.56 km) under the SSP585 scenario. This migration pattern indicates that *A. carmichaelii* is likely moving toward “climate refugia,” such as high-altitude and topographically complex areas (Hengduan Mountains or periphery of the Yunnan-Guizhou Plateau), to alleviate thermal stress [30]. Similar high-altitude migrations have also been observed in *Coptis chinensis* Franch and *Senna obtusifolia* (L.) H. S. Irwin & Barneby [15,34]. Furthermore, the migration toward elevated areas is conducive to reduce human acitivities, and thereby intensifing the decrease in suitable haibtats. This dual-driven habitat loss emphasizes the necessity of strengthening conservation strategies for wild populations of *A. carmichaelii* and highlights the importance of prioritizing high-altitude areas for future artificial introduction and standardized cultivation [24].

### 3.4. Research Limitations

Despite parameter optimization and effective predictive performance of the MaxEnt model, several limitations remain. First, the study deliberately restricted the modeling range to China, given China’s status as the biodiversity center for *Aconitum* species. Future studies should incorporate occurrence records from Vietnam to better understand the distribution shift across Asia. Second, this study used occurrence records collected after the year 2000 to represent current habitat. This approach might create temporal discrepancies with the climatic data (1970–2000). Future studies should improve data collection by expanding the temporal range (1970–present). Third, occurrence records from artificially cultivated populations of *A. carmichaelii* could contribute to the elevated significance of HFP. Future research should emphasize wild resource sites. Fourth, pollinator interactions, specifically between *A. carmichaelii* and bumblebees, influence reproductive processes. This study was limited to the effects of environmental variables on distribution under climate scenarios. Future research should integrate multi-species distribution models to assess spatio-temporal niche overlap and migration synchrony between *A. carmichaelii* and its pollinators, clarifying impacts of distribution shifts on population reproduction. Lastly, since single-model approaches carry structural uncertainty, future studies should adopt ensemble modeling frameworks, incorporating diverse machine learning algorithms (e.g., Random Forest and Generalized Additive Models) to cross-validate habitat predictions and minimize algorithm-specific biases.

## 4. Data and Methods

### 4.1. The Full Roadmap on Which Analyses Were Based Is Summarized in Figure 8

The analytical process of this study consisted of four stages. First, occurrence records were collected and environmental variables were screened. Second, the MaxEnt model was optimized. Third, the dominant environmental variables shaping the distribution of *A. carmichaelii* were identified, and its current and future distributions were projected. Finally, the ecological roles of these dominant variables, the projected distributional shifts, and the study’s limitations were discussed.

**Figure 8:**
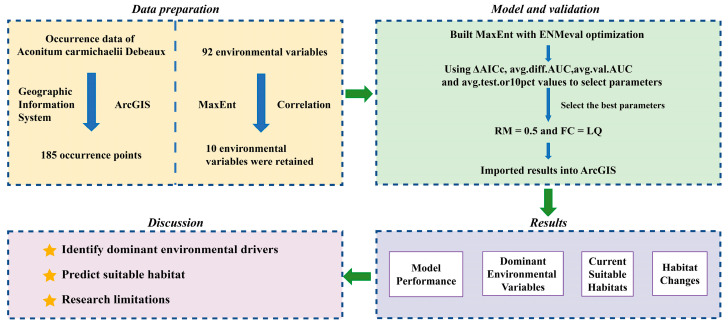
The roadmap used in this study.

### 4.2. Study Species

According to Flora of China, *A. carmichaelii* is a perennial herbaceous flowering plant distributed throughout southwestern, southern, southeastern, and northern China, as well as northern Vietnam. The species typically thrives on grassy slopes, shrublands, and stream edges at low to medium altitudes. Its stems are approximately 1 m tall, and it flowers between September to October with blue-purple sepals. Xiao et al. [17] reported the ecological characteristics of *A. carmichaelii* in Sichuan Province, where the species occurs at elevations of 800–1000 m, with a mean annual temperature below 15 °C, annual precipitation exceeding 800 mm, and mean annual sunshine duration exceeding 1000 h. Diester-diterpene alkaloids and monoester-diterpene alkaloids are composed of the main active Aconitum-type alkaloids in the lateral roots of *A. carmichaelii* [35,36]. The growth, health, and alkaloid production of *A. carmichaelii* are closely linked to its interactions with microorganisms; specifically, certain bacterial genera positively correlate with alkaloid accumulation [35]. Furthermore, root endophytic bacterial communities in *A. carmichaelii* exhibit regional variations, likely driven by differences in soil nitrogen, organic matter, and temperature [35]. Conversely, root rot caused by *Fusarium* fungi is a primary disease severely limiting cultivation yields [31].

### 4.3. Occurrence Records of A. carmichaelii

**Figure 9:**
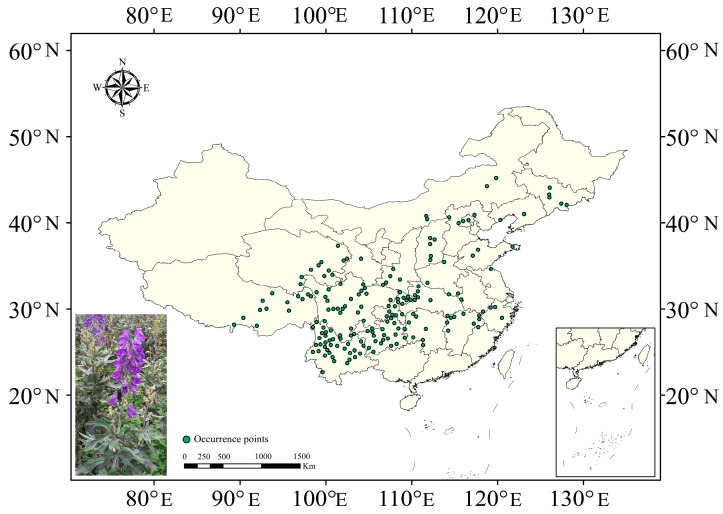
Occurrence records of *A. carmichaelii* in China.

Occurrence records from 1 January 2000 to 25 September 2025 for *A. carmichaelii* were collected from the Chinese Virtual Herbarium (https://www.cvh.ac.cn/ accessed on 25 September 2025), Global Biodiversity Information Facility (https://www.gbif.org/ accessed on 25 September 2025) as well as field investigations and literature survey. To ensure data quality and model accuracy, raw occurrence records were rigorously filtered. Records with ambiguous taxonomy, inaccurate geographic coordinates, or duplicates were excluded. Spatial thinning was performed in ArcGIS to reduce sampling bias and spatial autocorrelation. A buffer grid of 20 × 20 km was created, retaining only one occurrence record per grid cell. After this screening, 185 high-quality occurrence points remained for subsequent modeling (Figure 9, Table S1).

### 4.4. Environmental Variables

Current (1970–2000) and future bioclimatic variables (Bio 1 to Bio 19), minimum temperature (Tmin1 to Tmin 12), maximum temperature (Tmax1 to Tmax 12), average temperature (Tavg 1 to Tavg 12), precipitation (Prec 1 to Prec 12), solar radiation (Srad1 to Srad 12), and topgraphic (Elev) during four periods (2021–2040, 2041–2060, 2061–2080 and 2081–2100) were downloaded from the WorldClim database (https://www.worldclim.org/ accessed on 5 October 2025) at a 2.5’ spatial resolution. UV-B radiation variables (UVB 1–6) [37] were sourced from the gIUV database (https://www.ufz.de/gluv/ accessed on 10 October 2025), while topographic (slope and aspect) variables were obtained from Geospatial Data Cloud site, Computer Network Information Center, Chinese Academy of Sciences (http://www.gscloud.cn/ accessed on 10 October 2025). Anthropogenic factors were quantified using the Human Footprint dataset (http://www.earthdata.nasa.gov/ accessed on 10 October 2025), which integrates eight dimensions: built environment, population density, nighttime lights, cropland, pasture, roads, railways, and navigable waterways [38]. Edaphic factors were obtained from the Center for Sustainability and the Global Environment database (http://www.sage.wisc.edu/atlas/index.Php/ accessed on 13 October 2025). An initial set of 92 environmental variables was standardized to a 2.5 km resolution under a uniform geographic coordinate system [15,39]. Variables were reduced using Spearman correlation analysis; for variable pairs with |r| > 0.80, only the variable with a higher contribution was retained [15,34]. After rigorous selection based on the jackknife method and biological significance [39], 10 variables were chosen for model construction. These included seven bioclimatic variables, one HFP index, one edaphic factor, and one topographic factor (Table 3, Table S2).

### 4.5. MaxEnt Model

To avoid overfitting and excessive complexity from default parameters, the ENMeval package in R software(version 4.4.1) was used to optimize the MaxEnt model by adjusting FC and RM [13]. Specifically, the RM ranged from 0.5 to 4.0 in increments of 0.5, resulting in eight RM configurations, while five different FC types (L, LQ, LQH, LQHP, LQHPT) were evaluated. Among 40 parameter combinations, the optimal model was selected in case that Δ*AICc* equals 0. Model performance was further assessed using the avg.diff.AUC and the avg.test.or10pct [13,40]. Such optimization is especially crucial for species with limited sample sizes, as refined RM and FC significantly enhance discriminative accuracy [40].

Preliminary simulations tested different training/testing splits (70%/30%, 75%/25%, 80%/20%), and the optimal set was selected based on area under curve (AUC) values. The relative contributions of environmental variables were evaluated using the jackknife method [41]. Suitable habitats were assessed using 10,000 background points and 500 iterations [15]. Future predictions employed the BCC-CSM2-MR model from CMIP6 climate projections under scenarios SSP126, SSP245, SSP370, and SSP585, representing diverse greenhouse gas emission pathways [40]. The resulting suitability indices, ranging from 0 to 1, were classified into four categories: unsuitable (0–0.2), marginally suitable (0.2–0.4), moderately suitable (0.4–0.6), and highly suitable (0.6–1.0) [15].

## 5. Conclusions

Soil moisture and HFP significantly contribute to the distribution of *A. carmichaelii*, with highly suitable habitat located primarily within the Sichuan Basin and surrounding regions. The observed decline of highly suitable habitat for *A. carmichaelii* under all future scenarios is associated with the greenhouse effect together with human activities. The distribution shift towards high-altitude western regions provides both opportunity and challenge for sustainable cultivation and conservation strategy of *A. carmichaelii*.

## Figures and Tables

**Table 1:** AUC values under different ratios of training to test set.

	Training Set AUC Value
70% training set, 30% test set	0.892
75% training set, 25% test set	0.895
80% training set, 20% test set	0.892

**Table 2:** Quantitative predictions of *A. carmichaelii* distribution under current and future climatic scenarios (SSP126, SSP245, SSP370, SSP585).

Scenario	Period	Unsuitable Area (×10^5^ km^2^)	Marginally Suitable Area (×10^5^ km^2^)	Moderately Suitable Area (×10^5^ km^2^)	Highly Suitable Area (×10^5^ km^2^)
	Current	68.127	12.422	7.048	8.243
SSP126	2021–2040	68.88	14.952	8.786	3.220
	2041–2060	68.736	14.933	9.121	3.049
	2061–2080	67.869	15.107	9.053	3.810
	2081–2100	69.816	14.191	8.499	3.333
SSP245	2021–2040	69.749	14.156	8.715	3.219
	2041–2060	68.946	14.771	9.040	3.082
	2061–2080	70.126	14.075	8.477	3.162
	2081–2100	69.605	14.284	8.785	3.166
SSP370	2021–2040	69.135	14.648	8.769	3.287
	2041–2060	68.623	14.887	9.143	3.186
	2061–2080	69.948	13.932	8.411	3.548
	2081–2100	70.416	13.730	8.343	3.351
SSP585	2021–2040	69.023	14.785	8.769	3.262
	2041–2060	69.755	14.152	8.695	3.238
	2061–2080	69.589	14.426	8.692	3.133
	2081–2100	70.150	13.724	8.529	3.438

**Table 3:** Filtered environmental variables used in this study.

Abbreviation	Climate Variables	Unit
Bio 7	Temperature annual range	°C
Bio 15	Precipitation seasonality (coefficient of variation)	-
Prec 11	November precipitation	mm
Srad 05	Solar radiation in May	kJ m^−2^ day^−1^
Srad 07	Solar radiation in July	kJ m^−2^ day^−1^
Srad 10	Solar radiation in October	kJ m^−2^ day^−1^
Tmin 10	Minimum temperature in October	°C
Soilmoisl	Soil moisture	mm
Elev	Altitude	m
HFP	Human footprint	-

## Data Availability

Occurrence records were retrieved from the Chinese Virtual Herbarium (https://www.cvh.ac.cn/ accessed on 25 September 2025), Global Biodiversity Information Facility (https://www.gbif.org/ accessed on 25 September 2025) as well as field investigation and literature survey. The bioclimatic variables (Bio 1 to Bio19), minimum temperature (Tmin1 to Tmin 12), maximum temperature (Tmax1 to Tmax 12), average temperature (Tavg 1 to Tavg 12), precipitation (Prec 1 to Prec 12), solar radiation (Srad1 to Srad 12), and elevation (Elev) were retrieved from the WorldClim database (https://www.worldclim.org/ accessed on 5 October 2025). The global UV-B radiation (UVB 1 to UVB 6) was from global UV-B radiation database (https://www.ufz.de/gluv/ accessed on 10 October 2025). The Soil factors (Soil Moisture, Soil Organic Carbon and Soil pH) were from Center for Sustainability and the Global Environment database (http://www.sage.wisc.edu/atlas/index.Php/ accessed on 13 October 2025). The Human Footprint was from Nasa database (www.earthdata.nasa.gov/ accessed on 10 October 2025). The slope and Aspect were from Geospatial Data Cloud site, Computer Network Information Center, Chinese Academy of Sciences (http://www.gscloud.cn/ accessed on 10 October 2025). The geographic coordinates (latitude and longitude) of each occurrence record are available in the Supplementary Materials.

## Supplementary Materials

The following supporting information can be downloaded at: https://www.mdpi.com/article/10.3390/plants15071067/s1, Table S1: The distribution points of the *Aconitum carmichaeli*; Table S2: Correlation analysis of environmental variables for *Aconitum carmichaeli*. Figure S1: The predicted distribution of *A. carmichaelii* under future climatic scenarios. (A–C) represents predicted distribution during 2041–2060, 2061–2080, and 2081–2100 periods under SSP126 scenario, respectively; (D–F) represents predicted distribution duriing 2041–2060, 2061–2080, and 2081–2100 period under SSP245 scenario, respectively; (G–I) represents predicted distribution during 2041–2060, 2061–2080, and 2081–2100 period under SSP370 scenario, respectively; (J–L) represents predicted distribution during 2041–2060, 2061–2080, and 2081–2100 period under SSP585 scenario, respectively.
